# Quantitative evaluation of variation in defoliation traits among sugarcane genotypes

**DOI:** 10.1371/journal.pone.0196071

**Published:** 2018-05-10

**Authors:** Youzong Huang, Heyang Shang, Yuzhi Xu, Hongtao Jiang, Shiqiang Xu, Muqing Zhang

**Affiliations:** 1 Guangxi Cooperation and Innovation Center of Sugar Industry, Guangxi University, Nanning, Guangxi, China; 2 State Key Lab of Conservation and Utilization of Agric-Biological Resources, Guangxi University, Nanning, Guangxi, China; College of Agricultural Sciences, UNITED STATES

## Abstract

Development of easily defoliating sugarcane varieties is urgently needed to facilitate efficient mechanical harvesting, reduce production costs, and increase sugar yield in China. In order to quantify the defoliation characteristics of sugarcane, we investigated eight traits in two field experiments with a range of sugarcane varieties at maturity. The length (LSR) and angle (ASR) of the sheath ruptured from the stalk, defoliation force (DF), and self-defoliation (SD) were the traits with the greatest contribution to the quantitative assessment of sugarcane defoliation based on a principal component analysis, which accounted for more than 75% of the cumulative variability. A small set of traits, namely SD, ASR, and DF measured at the 10^th^ leaf were selected as predictors. Using these predictors, 37 out of 38 sugarcane cultivars were correctly classified into three groups (easy, difficult and intermediate in terms of ease of defoliation) that had been clustered based on six traits. These simplified measurements will be applied to screen new lines in the sugarcane breeding program in China.

## 1. Introduction

Sugarcane has become a pillar industry in Guangxi Zhuang Autonomous Region, China. Currently, more than 95% of sugarcane is manually harvested in China, in contrast to some other countries where mechanical harvesting dominates. The labor cost of manual harvesting is about RMB 150 (about USD 23) per tonne of cane, which is about 25% of the total production cost.

Removal of dead leaves from cane stalks (defoliation) is a major task during harvesting, adding greatly to costs. Burning of sugarcane was done in the past because it reduced costs of manual harvest and was believed to benefit ratoon growth. However, this production practice has been brought into question due to adverse effects of pollution from the large quantities of soot and gases produced [1–9]. One way to address these problems and facilitate green-cane harvesting is the development of easy-defoliating cultivars, which have leaves that easily fall off naturally as the cane matures [10].

Sugarcane has a high leaf area index (LAI) and a high photosynthetic efficiency under strong sunshine [11]. There are a range of studies suggesting that sugarcane might develop and maintain more green leaf area than is required for obtaining high levels of productivity and sugar concentration [12–14], suggesting that it might be possible to incorporate easy-defoliating characteristics without adverse effects on total recoverable sugar. The assimilation capacity of sugarcane leaves is robust and flexible to adapt the carbon supply based on the sink demand, and to preferentially partition the available carbon to sucrose culm storage [15]. The previous reports showed that partial defoliation in the month of August increased cane yield by 26.1% and CCS (t/ha) by 29.3% with decrease in leaf area index by 42.2% [16] because partial defoliation might be resulted not only in increased radiation interception, but also in an increase in rate of assimilation per unit radiation flux density.

In easy-defoliating cultivars, the leaf sheath ruptures at the point of attachment to the node, causing early separation of the leaf from the stem. Breeders have found variation in easy-defoliating characteristics that correlates with amount of trash at harvest. In this study, we assayed eight traits associated with defoliation on 38 sugarcane genotypes at maturity. The resulting data were interpreted as a guide for sugarcane breeders to incorporate selection for easy-defoliation to develop new varieties suitable for green-cane harvesting.

## 2. Materials and methods

### 2.1. Plant materials and field experiments

Two field experiments were conducted at the Chongzuo Institute of Agricultural Sciences located in Wutan, Nanning, Guangxi, China (E108°32′, N22°56′ and altitude of approximately 78 m). The experimental design was a randomized complete-block design with three replicates. There were four rows for each genotype and each replicate. Each plot area was 44.0 m^2^, with the row length of 10.0 m and the row space of 1.1 m. A total of 30,000 two-bud setts per ha were planted from February to March 2014. The previous crop was sugarcane and the soil was fertile and well irrigated. Crop management followed that practiced in conventional commercial production for the region.

Experiment I contained eight sugarcane genotypes, including two with easy defoliation (GXU35377 and GXU34179) and six with difficult defoliation (GXU34140, GXU35080, GXU36075, FN1110, FN40, and GT31). Eight traits associated with defoliation were measured on 15 individual plants in each genotype. These traits were listed in Table 1 along with a description of each. Measurements of these traits were made from the basal node to the first leaf, *i*.*e*. the first leaf from the top of the stalk with a clearly visible dewlap.

**Table 1:** Descriptions and measurements of defoliation traits.

Defoliation trait	Description and measurement
Total internode number (TIN)	Internode number counted from the soil level up to the 1^st^ leaf
Self-defoliating number (SDN)	Number of leaves per stalk that fall off naturally as the cane matures
Self-defoliating rate (SDR, %)	$SDR=\frac{SDN}{TIN}\times 100$
Stem diameter (SD, cm)	Stalk diameter (cm) was measured on the middle of the plant
Length of sheath ruptured from stalk (LSR, cm)	Length (cm) of sheath ruptured from stalk
Angle of sheath ruptured from stalk (ASR, °)	Angle (°) of sheath ruptured from stalk
Defoliation force (DF, N)	The defoliation force (N) was measured when one end of the rope was tied to the sheath far end of the sheath and the other end was tied to the digital Push & Pull Tester, keeping the rope perpendicular to the sugarcane and then slowly pulling off the leaves and finally read the measured value (defoliation force).
Mean defoliation force (MDF, N)	Defoliation force (N) was averaged in all leaves of same genotype.

The 1^st^ leaf (+1) was defined as the leaf on the first clearly visible dewlap.

Experiment II was conducted with 38 sugarcane genotypes in the China National Regional Test Program to validate quantitative evaluation indexes of defoliation and their defoliation capacity [17–18]. The same eight agronomic traits were measured on an average of 15 individual plants in each genotype from the 10^th^ to the 16^th^ leaf.

### 2.2. Data analysis

Data from each experiment was analyzed separately following the procedures below.

#### 2.2.1. Variance analysis

The means of measurements on the plants in each plot were averaged prior to analysis. The measurements of each trait were first tested for normality with the Shapiro-Wilk’s test [19]. If the variables failed the normality test, then they were transformed prior to variance analysis. One-way variance analysis (ANOVA) with Duncan’s post-hoc multiple comparisons were used to assess significant differences among the tested sugarcane genotypes.

#### 2.2.2. Principal component and clustering analysis

Mean data of eight traits from all leaf positions in each genotype were used for principal component analysis (PCA) and hierarchical clustering analysis (HCA) by the SAS/STAT procedures PRINCOMP and CLUSTER, respectively. All data were standardized before analysis (the mean of the values for each variable was subtracted from each variable value and the result was divided by the standard deviation of the values for each variable). Euclidean distance for HCA between sugarcane genotypes was calculated using the flexible group average method [20]. Hierarchical cluster analysis was carried out in three methods. Method-I: based on the six screened defoliation traits (SDN, SDR, LSR, ASR, DF, and MDF from the 6^th^ to 16^th^ leaf); Method-II: based on the same six screened defoliation traits (SDN, SDR, LSR, ASR, DF, and MDF from the 10^th^ to 13^th^ leaf); Method-III: based on the three simplified defoliation traits (SDR, ASR_10_ and DF_10_ at the 10^th^ leaf). Stepwise discriminant analysis (SDA) was used to select the variables most useful in discriminating the samples from the above HCA clusters using the SAS/STAT procedure STEPDISC. The variable within the model that contributed least to the model as determined by the Wilk’s Lambda method and the significance level of F test at *Pr* ≤ 0.05 was removed from the model. Similarly, the variable outside the model that contributed most to the model was added. When no more steps could be taken, the number of traits in the model was reduced to its final form. The final traits in turn were subjected to discriminant analysis to develop models for discriminating the defoliation capacity of the sugarcane genotypes. Groups were separated using Tukey’s test and considered significantly different at *Pr* ≤ 0.05 (SAS V.9.1, SAS Institute, NC, USA).

## 3. Results

### 3.1. Quantitative evaluation for sugarcane defoliation

The eight sugarcane genotypes in experiment I had marked differences (*Pr* ≤ 0.001) for all traits (Table 2). GXU34140, GXU35080, and FN40 had the highest defoliation force (DF and MDF), the least self-defoliating leaves (SDR and SDN), and the shortest length of sheath ruptured from stalk (LSR), while GXU34179 and GXU35377 were opposite in these traits.

**Table 2:** ANOVA analysis for the agronomic traits associated with sugarcane defoliation.

Genotypes	TIN	SDN	SDR (%)	SD (cm)	LSR (cm)	ASR (°)	DF (N)	MDF (N)
GXU34179	22.2 ± 0.73 AB	1.8 ± 0.92 B	7.68 ± 3.9 B	31.24 ± 1.32 AB	6.63 ± 0.9 A	44.33 ± 18.8 B	8.85 ± 0.54 D	0.58 ± 0.04 B
GXU36075	23.4 ± 0.93 A	0 ± 0 B	0 ± 0 B	32.44 ± 0.97 A	4.99 ± 0.5 AB	10.66 ± 3.69 BC	26.06 ± 2.99 C	1.41 ± 0.11 B
FN40	21.2 ± 0.37 ABC	0 ± 0 B	0 ± 0 B	30.56 ± 1.2 AB	2.99 ± 0.29 BC	4.76 ± 2.68 BC	50.41 ± 2.86 B	3.11 ± 0.17 A
FN1110	21.6 ± 0.6 ABC	0 ± 0 B	0 ± 0 B	28.09 ± 0.59 AB	3.64 ± 0.43 BC	1.81 ± 0.4 C	20.92 ± 0.51 CD	1.27 ± 0.06 B
GT31	20.6 ± 0.68 BC	0.2 ± 0.2 B	1 ± 1 B	32.09 ± 1.14 A	4.9 ± 0.77 AB	14.04 ± 6.55 BC	16.56 ± 2.05 CD	1.09 ± 0.15 B
GXU34140	19 ± 0.32 C	0 ± 0 B	0 ± 0 B	27.46 ± 0.44 B	2.05 ± 0.43 C	1.68 ± 0.48 C	50.72 ± 5.81 B	3.63 ± 0.43 A
GXU35377	23.8 ± 0.37 A	7.2 ± 1.07 A	30.42 ± 4.75 A	30.84 ± 1.13 AB	7.2 ± 0.7 A	94.47 ± 18.61 A	8.54 ± 2.74 D	0.67 ± 0.17 B
GXU35080	22.4 ± 0.81 AB	0 ± 0 B	0 ± 0 B	30.52 ± 1.18 AB	1.25 ± 0.33 C	0.73 ± 0.23 C	66.69 ± 7.96 A	3.79 ± 0.35 A
Mean	21.78 ± 0.31	1.15 ± 0.41	4.89 ± 1.74	30.40 ± 0.43	4.21 ± 0.37	21.56 ± 5.82	31.09 ± 3.52	1.94 ± 0.21

Numbers within columns followed by different capital letters showed significance at the level of 0.01 probability level.

**Fig 1:**
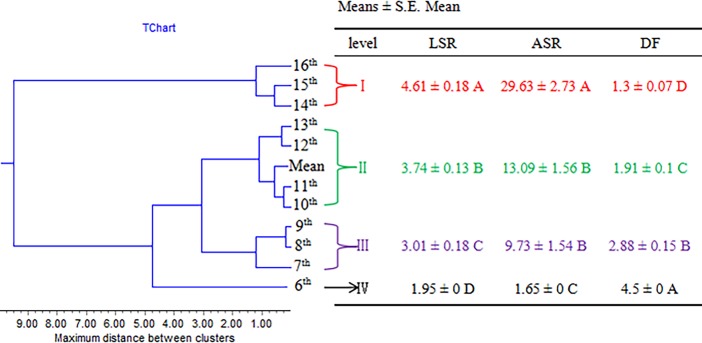
Dendrogram and summary of the categories clustered by HCA based on the quantitative defoliation traits (LSR, ASR and DF) measured at the 6^th^ to 16^th^ leaf. Different capital letters showed significant differences at 0.01 levels.

The first principal component (PC_1_) from PCA accounted for 63.1% of the total variance in the data set. The angle (ASR) and length (LSR) of sheath ruptured from the stalk were most highly positively related to this component, while defoliation force (DF) and mean defoliation force in each leaf (MDF) were negatively related (Table 3). The second principal component (PC_2_) accounted for 15.3% of the total variance, with the traits self-defoliating number (SDN) and self-defoliating rate (SDR) largely contributing to this component. Overall, these results indicated that the length and angle of the sheath ruptured from stalk (LSR and ASR), defoliation force (DF), and mean defoliation force (MDF) contributed the most to the variation in these data. In attempt to streamline measurements of the defoliation traits, we compared the differences of DF, LSR, and ASR from the 6^th^ leaf to the 16^th^ leaf. The results obtained by HCA were shown as a dendrogram (Fig 1), in which four well-defined clusters from the top 6^th^ leaf to the basal 16^th^ leaf were generated. Cluster I comprising the basal 14^th^ to 16^th^ leaves had the largest LSR and ASR, but the lowest DF, indicating easy defoliation. The cluster IV at the top 6^th^ leaf was opposite to cluster I, indicating difficult defoliation. LSR in cluster II (10^th^ to 13^th^) was significantly higher than that in cluster III (7^th^ to 9^th^), whereas the DF in cluster III was significantly greater than that in cluster II (*Pr* ≤ 0.01). The LSR, ASR, and DF values for Cluster II were similar to the means for all leaves, suggesting that LSR, ASR, or DF on each of the 10^th^ to 13^th^ leaves could represent the overall differences observed.

**Table 3:** Principal Components (PC) of the defoliation traits in sugarcane genotypes.

Defoliation trait	Principal component (PC)		
	1^st^ PC	2^nd^ PC	3^rd^ PC
Total internode number (TIN)	0.04	0.45	**0.73**
Self-defoliating number (SDN)	0.40	**0.90**	0.11
Self-defoliating rate (SDR)	0.40	**0.90**	0.11
Stem diameter (SD)	0.19	-0.06	**0.91**
Length of sheath ruptured from stalk (LSR)	**0.83**	0.35	0.33
Angle of sheath ruptured from stalk (ASR)	**0.74**	0.45	0.31
Defoliation force (DF)	**-0.93**	-0.29	0.00
Mean defoliation force (MDF)	**-0.93**	-0.21	-0.08
Characteristic root	5.05	1.22	0.90
Contribution rate (%)	63.13	15.28	11.26
Cumulative contribution rate (%)	63.13	78.41	89.67
Factor weight (ω)	0.70	0.17	0.13

### 3.2. Defoliation evaluation of sugarcane cultivars

In hierarchical cluster analysis, 38 sugarcane genotypes in Experiment II were classified on the basis of six traits, namely SDN, SDR, LSR_10_ and ASR_10_ at 10^th^ leaf, as well as DF and MDF from the 10^th^ to 16^th^ leaf (Method-I) and from the 10^th^ to 13^th^ leaves (Method-II). When compared by Method-1 to Method-2, four genotypes, YZ05-49, ROC22, LC03-182, and YG43, were clustered by both Methods-I and-II in category II (easy defoliation group), which showed the highest SDN and SDR, the greatest LSR and ASR, and the least DF and MDF. The category I (difficult defoliation group) was opposite to category II and included FN41, MT02-205, FN40 and LC07-500 by Method-I, but LC07-500 was absent by Method-II. All other genotypes were clustered into category III. These results indicated similar clustering using either Method-I or Method-II. To account for the correlation among the traits and to save time and cost of the measurements, SDR, ASR_10_ and DF_10_ at the 10^th^ leaf were used for the hierarchical cluster analysis and referred to as Method-III. The results by Method-III indicated that only MT02-205 and FN40 were also clustered into the difficult defoliation category as Method-I, but three cultivars (LC03-182, YR05-207 and ROC22) were clustered into the category of easy defoliation. The other 33 genotypes were clustered into category III, which could be divided into two sub-groups. The first sub-group in category III included 12 sugarcane cultivars, YR07-1433, YZ03-258, YR06-189, LC05-136, GZ07-538, LC07-500, FN41, YG43, LC05-129, LC03-1137, GT31 and DZ06-24, that had the larger ASR_10_ and DF_10_ at the 10^th^ leaf, but less SDR. The second sub-group included 21 sugarcane cultivars that had a higher DF_10_ at the 10^th^ leaf.

The results from stepwise discriminant analysis (SDA) indicated that SDR, ASR_10_ and DF_10_ at the 10^th^ leaf were selected for the discriminant function based on the Wilk’s Lambda and F value (*Pr* = 0.0000, *Chi* = 45.2). By using the three traits described above as predictors, 37 out of 38 sugarcane genotypes were correctly classified into HCA-clustered groups with an overall posteriori probability of 0.973 from all six defoliation traits, including SDN, SDR, LSR, ASR, DF, and MDF from the 6^th^ to 16^th^ leaf (Table 4). The overall results indicated that four sugarcane genotypes, ROC 22, LC03-182, YR05-207 and YZ05-49, were classed as easy to defoliate at a probability greater than 0.95. Three genotypes were classed as difficult to defoliate, FN40, FN41 and MT02-205. Seven other genotypes were also clustered as difficult defoliation with a lower probability, GT29, GZ07-538, LC05-136, LC07-500, YZ 03–258, YR06-189 and YZ04-2384.

**Table 4:** Defoliation categories (I: Easy defoliation; II: Difficult defoliation; III: Intermediate defoliation) and their posteriori probability of sugarcane genotypes determined by HCA (hierarchical cluster analysis) and SDA (stepwise discriminant analysis).

Genotypes	Method-I			Method-II			Method-III		
	HCA	SDA	Prob.	HCA	SDA	Prob.	HCA	SDA	Prob.
DZ03-83	III	III	0.88	III	III	0.92	III	III	0.87
DZ06-24	III	III	0.55	III	III	0.70	III	III	0.82
FN0335	III	III	0.98	III	III	1.00	III	III	0.75
FN07-2020	III	III	1.00	III	III	1.00	III	III	0.94
FN07-3206	III	III	0.97	III	III	1.00	III	III	0.94
FN38	III	III	0.97	III	III	1.00	III	III	0.72
FN40	II	II	1.00	II	II	1.00	II	II	1.00
FN41	II	II	0.95	II	II	1.00	III	*II	0.98
GT29	III	III	0.99	III	III	1.00	III	III	0.72
GT31	III	III	0.91	III	III	0.98	III	III	0.65
GT32	III	III	0.97	III	III	1.00	III	III	0.76
GZ07-538	III	III	0.77	III	III	1.00	III	III	0.90
LC03-1137	III	III	0.94	III	III	1.00	III	III	0.94
LC03-182	I	I	0.95	I	I	1.00	I	I	1.00
LC05-129	III	III	0.96	III	III	0.96	III	III	0.94
LC05-136	III	III	0.50	III	III	1.00	III	III	0.96
LC07-500	II	*III	0.70	III	III	1.00	III	III	0.95
LC07-536	III	III	0.91	III	III	0.81	III	III	0.83
MT01-77	III	III	0.97	III	III	0.95	III	III	0.93
MT02-205	II	III	0.98	II	II	1.00	II	II	0.99
ROC22	I	I	1.00	I	I	1.00	I	I	1.00
YG24	III	III	0.99	III	III	1.00	III	III	1.00
YG26	III	III	0.98	III	III	0.99	III	III	0.98
YG39	III	III	1.00	III	III	1.00	III	III	0.64
YG40	III	III	0.89	III	III	1.00	III	III	0.90
YG42	III	III	0.97	III	III	0.98	III	III	0.85
YG43	III	III	1.00	III	III	1.00	III	III	1.00
YG46	III	III	0.99	III	III	1.00	III	III	0.99
YR05-207	I	I	1.00	I	I	1.00	I	I	1.00
YR06-189	III	III	0.95	III	III	0.98	III	III	0.84
YR07-1433	III	III	0.69	III	III	0.72	III	III	0.97
YZ01-1413	III	III	0.64	III	III	0.85	III	III	0.98
YZ03-258	III	III	1.00	III	III	0.89	III	III	0.89
YZ05-49	I	I	1.00	I	I	1.00	III	*I	0.88
YZ06-80	III	III	0.67	III	III	0.90	III	III	0.84
YZ07-2384	III	III	0.82	III	III	0.93	III	III	0.63
YZ08-2060	III	III	0.53	III	III	0.95	III	III	0.89
YZ99-596	III	III	0.90	III	III	0.86	III	III	0.58

* The corrected categories by stepwise discriminant analysis.

## 4. Discussion

There are few if any published reports characterizing defoliation traits in sugarcane breeding programs. Even though many breeders may suggest that defoliation is easy to assess qualitatively, we would argue that while some genotypes may appear to be classed visually as easy to defoliate based on visual appearance, that this may not accurately portray ease of leaf removal. It is difficult to measure traits quantitatively on all leaves of a stalk due to labor costs, and an efficient protocol is required. Our results suggested that clustering based on measurements of SDR, ASR_10_ and DF_10_ at the 10^th^ leaf provided a very similar grouping of genotypes as using a much greater amount of data. This is the first report to quantitatively evaluate and characterize sugarcane defoliation in the sugarcane breeding program worldwide. PCA with the maximum variance rotated component matrix revealed that LSR, ASR, DF and SD were the traits with the greatest contribution to the quantitative assessment of sugarcane defoliation. SD was highly positively correlated with LSR and ASR, but negatively related to DF. It is difficult to investigate defoliating traits from all leaves due to the labor costs, especially LSR, ASR, and DF. In general, the leaves from the 1^st^ to the 5^th^ positions are difficult to remove from the stalk, so the LSR, ASR, and DF could not be accurately measured. However, the leaves from the 17^th^ position to the base of the sugarcane plant were lost or damaged during the farming practices. In our hierarchical cluster analysis, 38 sugarcane varieties were classified based on the six screened defoliation traits, including SDN, SDR, LSR, ASR, DF and MDF from the 6^th^ to the 16^th^ leaves (Method-I), and those from the 10^th^ to the 13^th^ ones (Method-II). The results obtained by HCA indicated that there were no substantial differences in the hierarchical cluster analysis using Method-I and Method-II. To account for the correlation among the traits and to improve the measurement efficiencies, three simplified traits (SDR, ASR_10_ and DF_10_ at the 10^th^ leaf) were used for the hierarchical cluster analysis (Method-III) and the stepwise discriminant analysis. By using the above three traits as predictors, 37 out of 38 sugarcane cultivars were correctly classified into HCA-clustered groups with an overall posteriori probability of 0.973 from all six screened defoliation traits from the 6^th^ to 16^th^ leaves (Table 4). Methods-III could save substantial labor costs. It takes 30 minutes per person to investigate all six defoliating traits in a single sugarcane plant, and 15 plants for each genotype would require 450 minutes per person. Therefore, this approach is not feasible and time economical. However, it would take 3 minutes per person to investigate three defoliating traits at the 10^th^ leaf, including SDR, ASR_10_, and DF_10_. Therefore, we concluded that SDR, ASR_10_ and DF_10_ at the 10^th^ leaf would be suitable and feasible to quantify sugarcane defoliation in our sugarcane breeding program in China.

Labor shortages have become a growing problem for sugarcane production in China. More than 50% of sugar cane acreage in the southwest sugarcane production region is located in the hilly areas where mechanized machinery is unsuitable, necessitating the use of hand labor for planting and harvesting. According to government statistics, labor costs for cane sugar increased 140% over the past five years, and will keep on rising annually. Sugarcane defoliation is becoming a serious problem for sugarcane harvesting due to the higher impurity rate, decrease in efficiency, and increase in cost in China [21]. Harvested trash is comprised principally of green and dry leaves, young immature apical portion of the shoots and soils. Furthermore, residues left in the field after harvesting green cane complicate the production of the ratoon crop. Thereby, the easy-defoliating traits offer the potential to facilitate green cane harvesting due to less green and dry leafy material at harvest. Breeders have found variations in easy-defoliating characteristics. Our results indicated that the defoliation capacity of 38 sugarcane genotypes from the National Regional Test Program for Sugarcane in China could be divided into three groups [17–18]. Four varieties, YZ05-49, ROC22, LC03-182 and YG43, were clustered in group I (easy defoliation group), which showed the highest self-defoliation (SDN, SDR), the greatest angle and length of sheath ruptured from the stalk (LSR and ASR) as well as the least defoliation force (DF and MDF). Four varieties were grouped into the difficult defoliation group, YZ03-258, MT02-205, FN40, and FN41. The other 30 genotypes were in moderate defoliation groups. ROC22 was introduced from Taiwan in 1998 and accounts for over 60% of the total sugarcane area in China [22]. ROC22 is an easy defoliation sugarcane cultivar. However, the progenies from ROC22 were different in their defoliation capacities (data not shown). Both GXU34176 (easy defoliation) and GXU34140 (difficult defoliation) were from the same cross of Co1001×ROC22, but their defoliation capacities were significantly different, suggesting that performance of parents did not well predict progeny performance in defoliation. The analysis of combining abilities indicated that it was difficult for breeders to improve the defoliation through the selection of parents, but there would also be no adverse effects of indirect selection for defoliation [23]. The transcriptomes of both GXU34176 (easy defoliation) and GXU34140 (difficult defoliation) at different growth stages have been elucidated in order to explore the genes and their molecular networks associated with the sugarcane defoliation and will be reported in the near future.

## Abbreviations

HCA: hierarchical cluster analysis
SDA: stepwise discriminant analysis
TIN: Total internode number
SDN: Self-defoliating number
SDR: Self-defoliating rate
SD: Stem diameter
LSR: Length of sheath ruptured from stalk
ASR: Angle of sheath ruptured from stalk
DF: Defoliation force
MDF: Mean defoliation force
LAI: leaf area index
PC: principal component
LSD: least significant difference
ANOVA: analysis of variance
SD: standard deviation
